# Enzymatic Hydrolysis of Oleuropein from *Olea europea* (Olive) Leaf Extract and Antioxidant Activities

**DOI:** 10.3390/molecules20022903

**Published:** 2015-02-11

**Authors:** Jiao-Jiao Yuan, Cheng-Zhang Wang, Jian-Zhong Ye, Ran Tao, Yu-Si Zhang

**Affiliations:** 1 Institute of Chemical Industry of Forest Products, CAF, Nanjing 210042, China; E-Mails: yuanjj88lhs@163.com (J.-J.Y.); yejianzhong@163.com (J.-Z.Y.); trmoon1949@126.com (R.T.); nmczhang@126.com (Y.-S.Z.); 2 Key and Open Laboratory on Forest Chemical Engineering, SFA, Nanjing 210042, China; 3 Key Laboratory of Biomass Energy and Matetial, Nanjing 210042, China; 4 Institute of New Technology of Forestry, CAF, Beijing 100091, China

**Keywords:** *Olea europea* L., hydroxytyrosol, oleuropein, enzymatic hydrolysis, hemicellulase

## Abstract

Oleuropein (OE), the main polyphenol in olive leaf extract, is likely to decompose into hydroxytyrosol (HT) and elenolic acid under the action of light, acid, base, high temperature. In the enzymatic process, the content of OE in olive leaf extract and enzyme are key factors that affect the yield of HT. A selective enzyme was screened from among 10 enzymes with a high OE degradation rate. A single factor (pH, temperature, time, enzyme quantity) optimization process and a Box-Behnken design were studied for the enzymatic hydrolysis of 81.04% OE olive leaf extract. Additionally, enzymatic hydrolysis results with different substrates (38.6% and 81.04% OE) were compared and the DPPH antioxidant properties were also evaluated. The result showed that the performance of hydrolysis treatments was best using hemicellulase as a bio-catalyst, and the high purity of OE in olive extract was beneficial to biotransform OE into HT. The optimal enzymatic conditions for achieving a maximal yield of HT content obtained by the regression were as follows: pH 5, temperature 55 °C and enzyme quantity 55 mg. The experimental result was 11.31% ± 0.15%, and the degradation rate of OE was 98.54%. From the present investigation of the antioxidant activity determined by the DPPH method, the phenol content and radical scavenging effect were both decreased after enzymatic hydrolysis by hemicellulase. However, a high antioxidant activity of the ethyl acetate extract enzymatic hydrolysate (IC_50_ = 41.82 μg/mL) was demonstated. The results presented in this work suggested that hemicellulase has promising and attractive properties for industrial production of HT, and indicated that HT might be a valuable biological component for use in pharmaceutical products and functional foods.

## 1. Introduction

Olive (*olea europaea* L.), mainly originated from Mediterranean region, is a famous woody oil tree used to produce virgin olive oil. Olive oil is the cardinal characteristic of the Mediterranean diet, serving as the principal source of dietary fat. Because it is rich in bioactive compounds (vitamins, flavonoids and polyphenols), it has been associated with lower rates of coronary heart disease (CHD), as well as reduced breast and colon cancer risk. Olive leaves also contain a wide variety of phenolic compounds, for example, oleuropein (OE), hydroxytyrosol (HT), tyrosol (T), cumaric acid, ferulic acid, caffeic acid, *etc.* These phenolic compounds have very good biological activity, *i.e.*, antioxidative activity [1,2], in addition to antimicrobial activity against *Helicobacter pylori*, *Campylobacter jejuni*, *Staphylococcus aureus* [3], and *Bacillus cereus*, *Escherichia coli* and *Salmonella enteritidis* [4]. They can play an important role in human diet and health to lower blood pressure, increase blood flow in the coronary arteries, and decrease the risk of cardiovascular disease [5]. Furthermore, olive leaf extract could be used as potentially safe natural additives for cosmetics, functional food and medicine. The small molecule HT, in particular, presents three free hydroxyl groups and presents strong anti-tumor bioactivity to inhibit the proliferation of human promyelocytic leukemia cells [6], human colon cancer HT-29 cells [7] and MCF-7 human breast cancer cells [8]. Researchers have shown that HT can be obtained by several chemical synthesis methods from tyrosol [9,10], 3,4-dihydroxyphenylacetic acid [11,12,13], 3,4-dimethoxybenzeneacetic acid [14], catechol [15], and protocatechuic aldehyde [16]. However, it is found that the yield of HT is very low due to the very long procedures. Besides, these chemical syntheses use a large amount of toxic agents and expensive catalysts. Due to its high cost and the low HT yield, so far HT is not offered on a pilot scale in the international market.

**Scheme 1:**
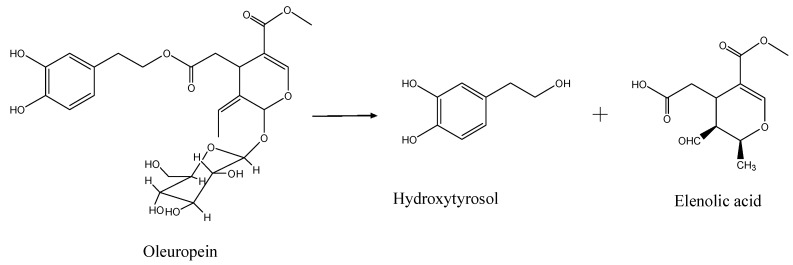
Oleuropein and its hydrolysis products.

HT exits widely in the plants in the form of OE glycosides, particularly in different parts of *Olea europea* (olive). HT is normally discarded in the waste water and olive pomace from the processing of olive fruits, so scientists have adopted adsorption resins to purify and enrich HT. However, due to the too low content of HT in olive fruit waste water, this method also does not provide HT on a large scale. Our studies had found more antioxidants in olive leaves than in olive oil [17], and the OE and HT content changed with different olive breeds and month of harvest [17,18]. Additionally, the level of HT was only 0.01%–0.8% [17], but that of OE was as high as 17% in olive leaf [18]. OE can be decomposed into HT and elenolic acid by different factors such as air, light, acid, base, oxidants, metal ions, high temperatures, etc. Scheme 1 presents the degradation process [19]. It was also noted that a lot of OE was decomposed into HT during storage, so the idea was proposed to prepare HT by degradation of OE from *Olea europea* (olive) leaf.

A number of reviews on the biotransformation for HT have appeared, providing a general idea on the biological synthesis of HT. Nucci and co-workers [19,20] have reported findings regarding olive leaves’ enzymatic hydrolysis properties. They used olive leaf extracts to produced large amounts of highly purified HT with a partially hyperthermophilic β-glucosidase immobilized on chitosan support (pH = 7.0, 60 °C) [20]. They exploited the capacity of chitin and chitosan of adsorbing polar molecules to purify HT. Espin’s group [21] reported the biotransformation of commercially available tyrosol into HT by mushroom tyrosinase, a polyphenol oxidase that catalyses hydroxylation of phenols to *o*-diphenols and *o*-quinones. Due to overoxidation to quinone, the reaction takes place under reducing conditions, in the presence of an excess of either ascorbic acid or NADH. The presence of the reducing agent allows the reversible formation of HT in a high yield and an environmentally-friendly procedure. Of course, *Pseudomonas aeruginosa* [22] and *Serratia marcescens* [23] were observed to transform tyrosol to HT. In addition, researchers also enriched HT by fungal species from olive oil wastewater [24] and liquid-solid olive oil wastes from two-phase olive processing [25].

Enzymatic hydrolysis not only involves mild conditions and simple operation methods, but also can obtain high purity product. Therefore, the present investigation was undertaken to study the enzymatic hydrolysis of natural OE (38.6% and 81.04% in olive leaf extract) to produce HT. Optimisation of the enzymatic hydrolysis was studied in order to improve the HT yield from olive leaf extract using RSM. Temperature, pH and enzyme quantity were fully examined by a Box-Behnken design. In addition, we investigated the phenol content and DPPH radical scavenging effect before and after the enzymatic hydrolysis.

## 2. Results and Discussion

### 2.1. Preparation for High Purity OE

**Figure 1:**
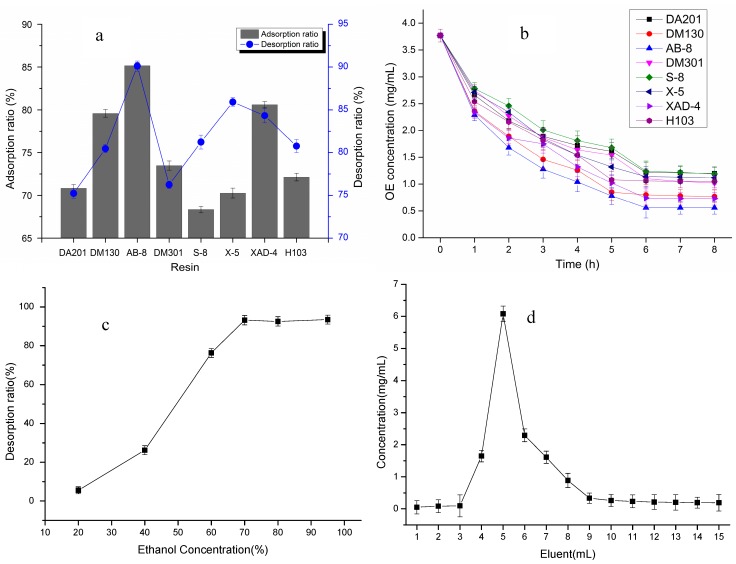
Adsorption, desorption performance, kinetic curves, elution effect of different ethanol concentration and dynamic adsorption curve of resins (*n* = 3). (**a**) adsorption and desorption performance of resins; (**b**) adsorption kinetic curves of resins; (**c**) elution effect of different ethanol concentration; (**d**) dynamic adsorption curve of resins).

38.6% OE olive extract sample were absorbed for 24 h by eight macroporous resins (DA201, DM130, AB-8, DM301, S-8, X-5, XAD-4, H103) , and the OE content in the adsorption liquids were measured by HPLC. Figure 1a shows that the adsorption performance was in the order: AB-8 (85.15%) > XAD-4 (80.59%) > DM130 (79.58%) > DM301 (73.47%) > H103 (72.13%) > DA201 (70.82%) > X-5 (70.26%) > S-8 (68.34%), and simultaneously, the desorption performance was in the order of AB-8 (90.12%) > X-5 (85.91%) > XAD4 (84.32%) > S-8 (81.23%) > H103 (80.76%) > DM130 (80.46%) > DM301 (76.23%) > DA201 (75.23%). AB-8 resin was thus the best for the adsorption and desorption of OE, and the adsorption and desorption rates (85.15%, 90.12%) were the highest, respectively. Figure 1b presents the adsorption kinetic curves of the resins. The resins all adsorbed OE quickly in 3 h, then the tendency became a little slower. The balance between adsorption and desorption occurred after 6 h. Furthermore, the adsorption capacity of the AB-8 resin was the highest among the macroporous resins, and the rate of adsorption was the quickest, so it could be concluded that the AB-8 resin was the optimal adsorption resin for OE purification. It was noted from Figure 1c that the desorption rate increased rapidly with ethanol concentration, but the desorbing effect of 70% ethanol was similar to 80% and 95% ethanol. This might be related to OE’s structure and chemical polarity. In view of the production cost, 70% ethanol was therefore the best eluant to desorb OE from AB-8 resin. The dynamic adsorption curve of AB-8 resin was presented in Figure 1d. The OE was concentrated in the 60–100 mL eluate.

Under these conditions, the content of OE can reach from 38.96% in the material to 65.59%, and then to 81.04% in the purified product after silica gel column chromatogram separation.

### 2.2. Enzymatic Hydrolysis of OE (Olive Leaf Extract) to HT

#### 2.2.1. Enzyme Selection for HT Biotransformation

HT is presented in the form of an OE aglycon and is obtained by hydrolysis from this glycoside during olive leaves’ storage and pressing due to the action of acidic, alkaline and/or enzymatic catalysis. It is reported that hyperthermophilic β-glucosidase is the key enzyme involved in the hydrolysis of OE, but the enzyme is very expensive. Cellulase is a cellulolytic enzyme, which comprises three different activities of endo-1,4-β-glucanase (EC.3.2.1.4), exo-1,4-β-D-glucanase (EC.3.2.1.74), β-1,4-glucosidase (EC.3.2.1.21). Cellulase and hemicellulase were used to decompose the cellulose and hemicellulose of plant cell walls. A report has shown that glucosidic bonds were hydrolyzed by hemicellulase [26].

**Figure 2:**
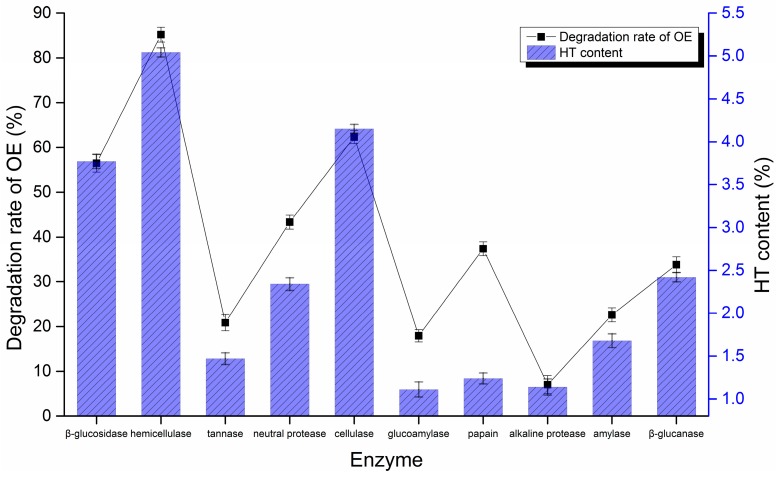
Enzymatic effect of different enzymes (*n* = 3).

Different enzymes (β-glucosidase, hemicellulase, tannase, neutral protease, cellulase, glucoamylase, papain, alkaline protease, amylase, β-glucanase) were used to hydrolyze OE to HT from 38.6% OE olive leaf extract. Figure 2 indicates that the best enzymatic hydrolysis performance corresponded to hemicellulase, which gives the highest OE degradation rate (81.21%) and HT content (5.04%). Cellulase (62.33%, 3.95%) and β-glucosidase (56.47%, 3.77%) were second to hemicellulase. These enzymes are known to play an important role in the degradation of the phenolic compound substrates during fungal bioprocessing. The tannase (20.87%, 1.47%), glucoamylase (33.81%, 2.42%) and alkaline protease (7.03%, 1.14%) had poor effects on the degradation of OE and production of HT. The use of hemicellulase enrichment for HT may prove useful in solving the problem of low concentrations of HT in olive leaf extract and the industrial technical problems relating to chemical hydrolysis of olive leaf extract. This process produces a natural and bioactive product from a vegetal source as opposed to a molecule obtainable only through chemical synthesis. The proposed enzymatic pre-treatment may prove useful not only for laboratory applications, but also for potential industrial applications of olive leaf extract. 

#### 2.2.2. Substrate for HT Biotransformation

**Figure 3:**
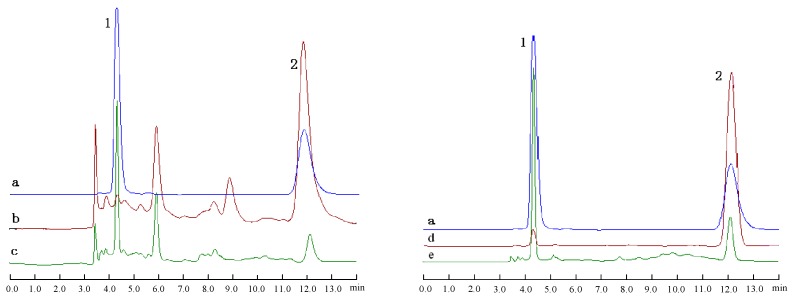
HPLC chromatograms of hydroxytyrosol and oleuropein mixed standard, olive leaf extract (38.6% OE, 81.04% OE) before and after enzymatic hydrolysis with hemicellulase.

Qualitative and quantitative analyses of HT and OE from olive leaf extract before and after enzymatic hydrolysis procedure were performed by HPLC. In Figure 3, 1 is HT and 2 is OE, with retention time of 4 min and 12.0 min, respectively. According to the retention time and their spectra, OE and HT content in olive leaf extract and enzymatic products were analysed with an external standard method.

(^a^ Hydroxytyrosol and oleuropein mixed standard samples; ^b^ olive leaf extract (38.6% OE); ^c^ enzymatic hydrolysate (38.6% OE); ^d^ olive leaf extract (81.04% OE); ^e^ enzymatic hydrolysate (81.04% OE); ^1^ hydroxytyrosol; ^2^ oleuropein).

Under the same enzymatic conditions, the degradation rate of OE was 82.59% and the HT content was enhanced to 5.62% for 38.6% OE olive leaf extract, however, the degradation rate of OE was 95.16% and the HT content was enhanced to 10.38% for 81.04% OE olive leaf extract. HPLC chromatograms of HT and OE before and after hydrolysis of the different substrates (38.6% OE and 81.04% OE olive leaf extract) are shown in Figure 3, and these profiles demonstrate an increase in the HT peak with a concomitant decrease of the OE peak after hydrolysis treatment. The HT content of enzymatic 38.6% OE hydrolysate was not higher than that of 81.04% OE samples. The high purity of OE in olive extract was beneficial for the biotransformation of OE into HT. It should be taken into account that 38.6% OE olive extract possesses 38.6% OE and 60% other impurities, including various compounds which may hinder the degradation process of OE to HT. In view of the degradation mechanism, the high purity OE in olive extract was used for biotransformation into HT.

### 2.3. Enzyme Hydrolysis from High Purity OE (81.04%) Olive Leaf Extract to HT

#### 2.3.1. Effect of pH

**Figure 4:**
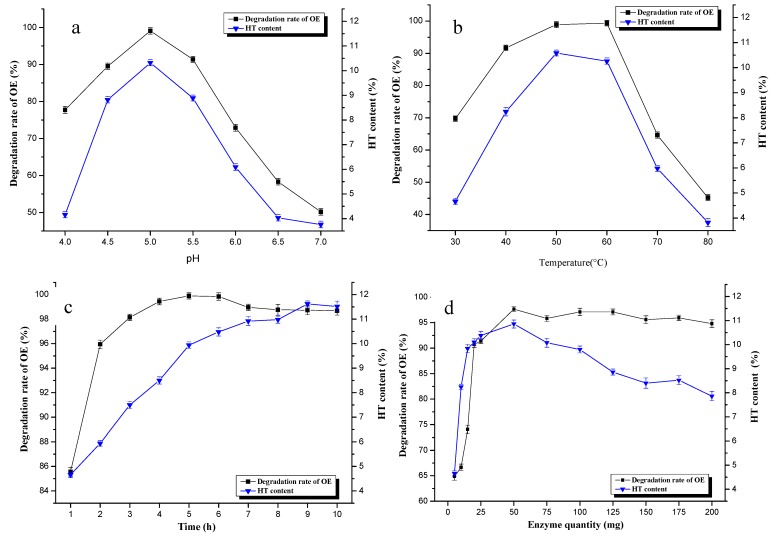
Effect of factor (**a**), pH; (**b**), temperature; (**c**), time; (**d**), enzyme quantity) on enzymatic hydrolysis (*n* = 3).

The effect of pH on the OE degradation rate and HT content is shown in Figure 4a. Enzymatic hydrolysis were carried out at different pH values (4.0, 4.5, 5.0, 5.5, 6.0, 6.5, 7.0), while the other parameters were fixed as follows: temperature of 60 °C, time of 6 h, and enzyme quantity of 50 mg. From the pH and OE degradation rate and HT content scatter diagram, the tendencies were very similar. They both increased in a steep manner when the pH ranged from 4.0 to 5.0, then decreased regularly as the pH increased. When pH = 5.0, the degradation rate of OE was 99.07%, and the HT content was 7.22%. Figure 4a shows that hemicellulase possesses enzymatic activity between 4.0 and 6.0, and the enzymatic efficiency is best at pH = 5.0. It was thus demonstrated that the enzymatic process was not suitable in more acidic or alkaline solutions.

#### 2.3.2. Effect of Temperature

Figure 4b presents the effect of temperature on the OE degradation rate and HT content. The enzymatic hydrolysis was carried out at different temperatures (30, 40, 50, 60, 70, 80 °C), while the other parameters were as follows: pH of 5, time of 6 h, and enzyme quantity of 50 mg. It was found that the OE degradation rate and HT content sharply increased with increasing temperature, reaching a peak value at 50 °C. The optimum temperature for HT content from hemicellulase hydrolysis was therefore 50 °C, and the degradation rates of OE at 50 and 60 °C were nearly the same (Figure 4b). With further temperature increased the HT content and the OE degradation rate were shown to decline sharply. This might be explained by the fact that a high temperature led to a decrease in enzyme activity. The optimal temperature was 50 °C, which was quite a high temperature.

#### 2.3.3. Effect of Bioconversion Time

Figure 4c show s the time course of HT formation during these reactions. Enzymatic hydrolyses were carried out for different times (1, 2, 3, 4, 5, 6, 7, 8, 9, 10 h), while the other parameters were fixed as follows: pH of 5, temperature of 60 °C, and enzyme quantity of 50 mg. The OE degradation rate increased to reach 99.8% at 5 h. However, this rate decreased from this time, and then stabilized at 98% for the whole period of time. It was found that the HT content presented a rapid increase from 1 h to 6 h, but the content did not increase much from 7 h to 10 h. Therefore 6 h was the best time for the hydrolysis process. The results could be explained by the fact that OE was first degraded into OE aglycon by the enzyme, then HT was formed from this glycoside over a long time. The second step did not produce HT fully in a short time.

#### 2.3.4. Effect of Enzyme Quantity

Enzymatic hydrolyses were carried out with different quantityies of enzyme (5, 10, 15, 20, 25, 50, 75, 100, 125, 150, 175, 200 mg) while other parameters were as follows: pH of 5, temperature of 60 °C, and time of 6 h. From the graph of the effect of enzyme quantity on enzymatic hydrolysis (Figure 4d), the highest degradation rate of OE (97.67%) was found for the 50 mg enzyme quantity. Additionally, the degradation rate of OE was stable after the 50 mg enzyme biotransformation process. HT content increased sharply with the initial 50 mg and decreased in a less steep behavior with increasing enzyme quantity.

#### 2.3.5. Optimization of Enzymatic Hydrolysis by RSM

Based on the results of the single factor experiments, the effect of time on the HT content was not significant, therefore a 17-run Box-Behnken design (BBD was used to optimize the three independent variables, including X_1_ (pH), X_2_ (temperature) and X_3_ (enzyme quantity). In order to study the combined effect of these factors, investigations were performed to use different combinations of the physical parameters. Table 1 showed the experiments design and dependent variable (HT content).

The analysis of variance was shown in Table 2 and Table 3. As shown in Table 2, the model F-value of 14.98 implied the model was significant. However, the lack of fit relative to the pure error was significant, so the statistical data presented in Table 3 was obtained again by manual optimization of the model.

**Table 1:** Box-Behnken experimental design and corresponding HT content.

No.	*X*_1_ pH	*X*_2_ Temperature/°C	*X*_3_ Enzyme Quantity/mg	*Y* HT Content/%
1	−1	−1	0	10.02
2	1	−1	0	9.51
3	−1	1	0	9.82
4	1	1	0	9.98
5	−1	0	−1	9.46
6	1	0	−1	9.82
7	−1	0	1	9.53
8	1	0	1	9.98
9	0	−1	−1	10.14
10	0	1	−1	9.87
11	0	−1	1	9.07
12	0	1	1	10.92
13	0	0	0	11.24
14	0	0	0	11.16
15	0	0	0	11.21
16	0	0	0	11.03
17	0	0	0	11.17

**Table 2:** Variance analysis of items in regression equation.

Sources of Variation	Sum of Squares	*df*	Mean Square	*F* value	*P* value
model	8.02	9	0.89	14.98	0.0009
*X*_1_	0.026	1	0.026	0.44	0.5263
*X*_2_	0.43	1	0.43	7.19	0.0315
*X*_3_	0.00513	1	0.005513	0.093	0.7697
*X*_1_*X*_2_	0.11	1	0.11	1.89	0.2120
*X*_1_*X*_3_	0.00202	1	0.00202	0.034	0.8589
*X*_2_*X*_3_	1.12	1	1.12	18.89	0.0034
*X*_1_^2^	2.80	1	2.80	47.13	0.0002
*X*_2_^2^	1.11	1	1.11	18.66	0.0035
*X*_3_^2^	1.77	1	1.77	29.77	0.0009
residual	0.42	7	0.059		
lack of fit	0.39	3	0.13	20.12	0.0071
pure error	0.026	4	0.00647		
total	8.43	16			
R^2^	0.9506				
R_adj_^2^	0.8872				
Adeq precision	10.141				

**Table 3:** Variance analysis of items in regression equation by manual optimization.

Sources of Variation	Sum of Squares	*df*	Mean Square	*F* Value	*P* Value
model	8.40	11	0.76	113.34	<0.0001
*X*_1_	0.16	1	0.16	24.34	0.0043
*X*_2_	0.62	1	0.62	92.62	0.0002
*X*_3_	0.005513	1	0.005513	0.82	0.4072
*X*_1_*X*_2_	0.11	1	0.11	16.65	0.0095
*X*_1_*X*_3_	0.002025	1	0.002025	0.30	0.6071
*X*_2_*X*_3_	1.12	1	1.12	166.74	<0.0001
*X*_1_^2^	2.80	1	2.80	416.06	<0.0001
*X*_2_^2^	1.11	1	1.11	164.76	<0.0001
*X*_3_^2^	1.77	1	1.77	262.78	<0.0001
*X*_1_^2^ *X*_2_	0.21	1	0.21	31.83	0.0024
*X*_1_ *X*_2_^2^	0.17	1	0.17	24.96	0.0041
residual	0.034	5	0.006739		
lack of fit	0.007812	1	0.007812	1.21	0.3335
pure error	0.026	4	0.00647		
total	8.43	16			
R^2^	0.9960				
R_adj_^2^	0.9872				
Adeq precision	29.880				

The HT content obtained at various levels of the three independent variables (X_1_, X_2_, and X_3_) were subjected to multiple regression to yield a second-order polynomial equation, as follows:

(1) $$Y=11.16+0.20X_{1}+0.39\;X_{2}+0 .026X_{3}+0.17X_{1}X_{2}+0.023X_{1}X_{3}+0.53X_{2}X_{3}-0.82{X_{1}}^{2}-0.51{X_{2}}^{2}-0.65{X_{3}}^{2}-0.33{X_{1}}^{2}X_{2}-0.29X_{1}{X_{2}}^{2}$$

where Y represented the HT content (%) and X_1_, X_2_ and X_3_ represented pH, temperature and enzyme quantity, respectively. According to the regression model equations, the fitting coefficient of three variables showed values of 0.39 > 0.20 > 0.026, implying that the X_1_ (pH) and X_2_ (temperature) were the main variables in the enzymatic hydrolysis of olive leaf extract.

The statistical parameters obtained from the analysis of variance for the manual optimization models are given in Table 3. The model F-value of 113.34 and *p* < 0.001 implied the model was significant, and the models could predict the real experimental data. Meanwhile, the value of the determination coefficient (*R*^2^ = 0.9960) and the adjusted determination coefficient (*R*_adj_^2^ = 0.9872) also confirmed that the model was statistically significant. Additionally, the lack of fit was not significant relative to the pure error. For the pure error, *p* > 0.05, implied that the calculated values could be fit with the experimental values. Adep precision measured the signal to noise ratio and a ratio greater than 4 is desirable. Thus, the ratio of 29.880 indicated an adequate signal. In conclusion, the model was found to be adequate for navigating the design.

The *p*-value is used as a tool to check the significance of each coefficient, which in turn indicates the interaction strength between each independent variable. It could be observed that the coefficient of X_2_X_3_, X_1_^2^, X_2_^2^ and X_3_^2^ were highly significant (*p* < 0.001). Meanwhile, the coefficient of X_1_, X_2_, X_1_X_2_, X_1_^2^X_2_ and X_1_X_2_^2^ were found to be significant (*p* < 0.05). Moreover, the coefficient of X_3_ and X_1_X_3_ were found non- significant (*p* > 0.05). The X_1_ and X_2_ variables had a significant effect on enzymatic hydrolysis. For the X_1_X_2_ interaction, *p* < 0.05, implied that the pH was related to temperature in the enzymatic process. Moreover, for the X_2_X_3_ interaction, *p* < 0.001, implied that the temperature was closely related to enzyme quantity in the enzymatic process.

**Figure 5:**
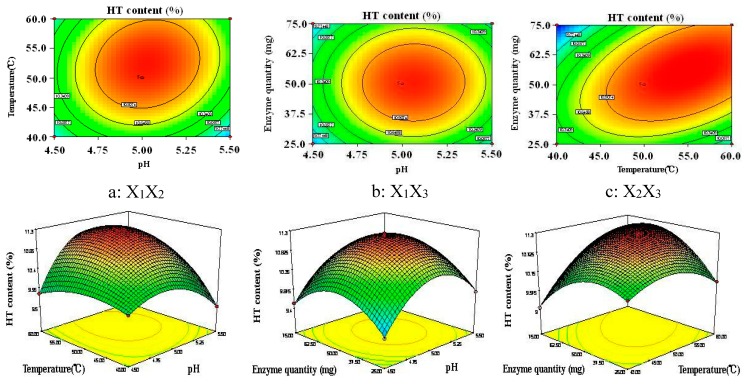
Contour plots and 3D-response surface plots showing the interactive effects of pH and temperature (**a**, X_1_X_2_), pH and enzyme quantity (**b**, X_1_X_3_), temperature and enzyme quantity (**c**, X_2_X_3_). (X_1_, pH; X_2_, temperature; X_3_, enzyme quantity).

The contour plots and 3D response surface plots, obtained by the Design Expert software, shown in Figure 5, are the graphical representations of the regression. In the contour plots and 3D response surface plots, the HT content was obtained along with two continuous variables, while the other variable were fixed constant at its 0 level. In the two figures, the maximum predicted value indicated by the surface was confined in the smallest ellipse in the contour diagram. Elliptical contours were obtained when there was a perfect interaction between the independent variables.

The HT content affected by the pH and temperature is seen in Figure 5a when enzyme qua ntity is maintained at the 0 level. It was obvious that the HT content increased as the pH was extended from 4.5 to 5. Subsequently, the HT content gradually decreased. The HT content also increased as temperature increased from 40 to 55 °C; the HT content slightly reduced after temperature of 55 °C. Figure 5b shows the HT content for varying pH and enzyme quantity. The round contour indicated that the interaction effect between these factors was not significant. This was in agreement with the p-value of X_1_X_3_. The maximum HT content was obtained when the pH and enzyme quantity were 5 and 55 mg, respectively. The HT content affected by temperature and enzyme quantity is shown in Figure 5c, when the variable (pH) is fixed at 0 level. The maximum HT content was obtained when the temperature and enzyme quantity were 55 °C and 55 mg, respectively.

After the BBD of enzymatic hydrolysis of olive leaf extract, the optimal enzymatic conditions for achieving maximal yield of HT content obtained by the regression were as follows: pH 5.06, temperature 54.80 °C and enzyme quantity 55.45 mg. The predicted yield value was 11.28%. Considering the practical experiments and production, the optimal parameters were adjusted to pH 5, temperature 55 °C and enzyme quantity 55 mg. Under this conditions, the experimental yield of HT was 11.31% ± 0.15% (*n* = 3), and the degradation rate of OE was 98.54%.

Ghayth *et al*. [27] explored the possibility of obtaining HT in high yield from 2-phase Chemlali olive waste by an acid/alkaline chemical treatment. The results showed that a high amount of hydroxytyrosol (1360 mg/kg of fresh 2-phase olive pomace) was obtained using water bath after treatment at 80 °C for 90 min with 1 M of H_3_PO_4_, whereas the alkaline conditions (1085.8 mg/kg) were not very suitable.

Bu *et al*. [28] examined olive leaves extract hydrolysis by hydrochloric acid and β-glucosidase, respectively, to prepare HT. The hydrochloric acid method was better (7.83%) than enzymatic hydrolysis (3.91%), and the HT content was also calculated with the ratio of HT and olive leaf extract weight. The methods and idea were very similar to our experiments, but the results were lower than our results using enzymatic methods. This might be due to using the low content of OE (25%) in olive leaf extract and low enzyme activity of β-glucosidase (25 U/mg).

Additionally, according to Briante’s [19] schematic OE reaction pathway, OE would be degraded into OE aglycon with hyperthermophilic β-glucosidase, then HT could be obtained in the presence of esterase enzymes. In our results showing OE high degradation rate and low yield of HT, this can be also explained by the action of hemicellulase only for separation of the glucoside, but with partial ester links still present in the OE, which leads to the production of HT, so the synergistic effect of hemicellulase and esterase enzyme will be investigated in future work.

### 2.4. Antioxidant Activity

Total phenols content of the 38.6% OE sample and 81.04% OE sample before and after enzymatic hydrolysis are shown in Table 4. 

**Table 4:** Phenols content, hydroxytyrosol (HT) concentration and radical scavenging effect (mean value ± SD, *n* = 3).

Samples	Phenol/%	HT/%	IC_50_/μg·mL^−1^
38.6% OE sample	1.46 ± 0.03	0.25 ± 0.01	82.31 ± 1.26
38.6% OE sample enzymatic hydrolysate	0.38 ± 0.01	2.81 ± 0.08	382.83 ± 0.72
81.04% OE sample	2.96 ± 0.07	3.88 ± 0.08	9.43 ± 0.08
81.04% OE sample enzymatic hydrolysate ^1^	0.46 ± 0.01	5.64 ± 0.03	216.40 ± 0.54
81.04% sample ethyl acetate extracted enzymatic hydrolysate ^2^	2.89 ± 0.06	19.36 ± 0.31	41.82 ± 0.17
BHT			30.27 ± 0.55
Vc			3.31 ± 0.06
OE			4.97 ± 0.08
HT			1.07 ± 0.02

^1^ The HT content before hydrolysis treatment was HT content/olive leaf extract; ^2^ The HT content after hydrolysis treatment was HT content/hydrolysate.

Phenol content was decreased after enzymatic hydrolysis, but the HT content was increased. Phenol content in the 38.6% OE sample enzymatic hydrolysate decreased from 1.46% to 0.38%, and in the 81.04% OE sample enzymatic hydrolysate it decreased from 2.96% to 0.46%. However, HT increased to 2.81% (38.6% OE hydrolysate) and 5.64% (81.04% OE hydrolysate), respectively. Using ethyl acetate extraction the phenol content was up to 2.89% and the HT content was also up to 19.36%.

In this study, the antioxidant activity of 38.6% sample, 81.04% sample, 38.6% sample enzymatic hydrolysate, 81.04% sample enzymatic hydrolysate, 81.04% sample ethyl acetate extracted enzymatic hydrolysate, BHT, Vc, and HT are measured by the DPPH free radical scavenging method and the corresponding IC_50_ values are shown in Table 5. BHT and Vc as positive reference compounds both showed strong antioxidant activity with an IC_50_ value of 30.27 and 3.31 μg/mL. Moreover, the antioxidant activity of OE and HT were also high, and the corresponding IC_50_ values were 4.97 and 1.07 μg/mL, respectively. The antioxidant activities were in the order: HT (1.07) > Vc (3.31) > OE (4.97) > BHT (30.27). However, the antioxidant activity of 38.6% and 81.04% sample enzymatic hydrolysates (IC_50_, 382.83, 216.40 μg/mL) were worse than the samples before the enzymatic process (IC_50_, 82.31, 9.43 μg/mL). This could be explained by the fact that the composition of the enzymatic hydrolysates was complex, with many neutral materials such as OE glycosides. However, the IC_50_ value of the 81.04% sample enzymatic hydrolysate after ethyl acetate extraction was 41.82 μg/mL. The antioxidant activity of phenolic extracts depends on the qualitative characteristics of phenolic profile and not just on the total phenolic content, so enzymatic hydrolysates still needed to be purified in order to obtain high antioxidant activity compounds (HT).

From these results we had noted the very low antioxidant activity of enzymatic hydrolysate (IC_50_ = 216.40, 382.83 μg/mL) and high antioxidant activity of the ethyl acetate extracted enzymatic hydrolysate (IC_50_ = 41.82 μg/mL). This meant that the OE was partly decomposed into the OE aglycone, but not completely transformed into HT in the enzymatic process, and most phenolic compounds having high antioxidant activity properties were not separated and purified during the enzymatic procedure.

## 3. Experimental Section

### 3.1. Materials

Olive leaf extract, powder, (OE, 38.6%) was purchased from Xi’an Bioengineer Company (Xi’an, China). Olive leaf extract (OE, 81.04%) was obtained with resin purification in the laboratory. Butylated hydroxytoluene (BHT), ascorbic acid (Vc), Folin–Ciocalteu phenol reagent and gallic acid were purchased from Sigma Chemicals (St Louis, MO, USA). HT and OE standard were also purchased from Sigma Chemicals. 1,1-diphenyl-2-picrylhydrazyl (DPPH) was purchased from Aladdin Chemicals (Shanghai, China). The ten tested enzymes (β-glucosidase, hemicellulase, tannase, neutral protease, cellulase, glucomylase, papain, alkaline protease, amylase, β-glucanase, Table 5) were all purchased from Aladdin Chemicals. Eight macroporous resins (DA201, DM130, AB-8, DM301, S-8, X-5, XAD-4, H103) were all purchased from Aladdin Chemicals, too.

**Table 5:** The specific information of the different enzymes.

Enzymes	pH	Temperature/°C	Enzyme Activities/u·mg^−1^	Form
β-glucosidase	5	37	30	powder
hemicellulase	4.0–5.5 (5)	45–60 (60)	20	powder
tannase	4.5–6 (5)	45–50 (50)	10	powder
neutral protease	5.5–8 (7)	45–50 (50)	13	powder
cellulase	6–7 (6)	45	10	powder
glucoamylase	4.0–4.5 (4.5)	60	5	powder
papain	6–7 (6)	55–65 (60)	80	powder
alkaline protease	8	50	20	powder
amylase	4.2	60	3	powder
β- glucanase	6.0–6.5 (6)	50–55 (50)	20	powder

The content in parentheses was the enzymatic hydrolysis conditions of each enzyme used in the experiments.

### 3.2. Preparation of the High Purity OE

Olive leaf extract was purified by adsorbed on macroporous resin. Eight macroporous resins (DA201, DM130, AB-8, DM301, S-8, X-5, XAD-4, H103) were used to purify OE, and the adsorption characteristics were also compared. The purification of OE include pretreatment of resins, saturated static adsorption, static adsorption kinetics, static desorption for different concentrations of the eluent and dynamic adsorption. The specific steps were as follows:

#### 3.2.1. Pretreatment of Resins

The resins were all soaked in ethanol for 24 h, then added to the column. Resins were washed with ethanol at a flow rate of 3 mL/min till the effluent was not turbid, then washed with water at the same rate till there was no ethanol in the effluent. Furthermore, the resins were washed with 5% HCl solution at 3 mL/min flow rate, and soaked 2 to 4 h. Then resins were washed with water again till the pH value of outflow water was neutral at the same rate. Moreover, resins were washed with 2% NaOH solution at 3 mL/min flow rate, and soaked 2 to 4 h, and then resins were washed with water till pH value of outflow water was neutral at the same rate again.

#### 3.2.2. Saturated Static Adsorption

Wet resin (DA201, DM130, AB-8, DM301, S-8, X-5, XAD-4, H103, respectively, 5 g) was placed in a 100 mL conical flask, and aqueous sample solution (1 g olive leaf extract in 50 mL water, 50 mL) was added for static adsorption. The flasks were shaken for 24 h at room temperature, and the solutions were filtered to collect the adsorption liquid. Then the OE content was determined by HPLC, and the saturation adsorption quantity was calculated.

#### 3.2.3. Static Adsorption Kinetics

Wet resin (DA201, DM130, AB-8, DM301, S-8, X-5, XAD-4, H103, respectively, 10 g) was placed in a 100 mL conical flask, and aqueous sample solution (50 mL) was added for static adsorption. The flask was maintained in a shaking state for 8 h, and 0.5 mL liquid was sampled per 1 h during the shaking process. Then the OE content of each sample was determined by HPLC, and the static adsorption curves were drawn.

#### 3.2.4. Static Desorption for Different Concentrations of the Eluent

Wet AB-8 resin (10 g) was placed in a 100 mL conical flask, and aqueous sample solution (50 mL) was added for static adsorption. The flasks were shaken for 24 h at room temperature, and the solution was filtered to collect the adsorbed resin. Then the residual resin were added to 50 mL 20%, 40%, 60%, 70%, 80%, 95% aqueous ethanol solution, respectively. The resin were desorbed for 12 h in the shaking state and the solutions were filtered to collect the desorption liquid. Then the OE content was determined by HPLC.

#### 3.2.5. Dynamic Adsorptions

Wet AB-8 resin was packed into a column (30 cm × 2.0 cm), and sample solution was added. The resin column was washed with water until the effluent was not turbid, then eluted with a certain concentration of ethanol. The eluted liquid was concentrated using a rotary evaporator, then the concentrate was diluted in a 100 mL volumetric flask with methanol, and the OE content was determined by HPLC.

### 3.3. Enzymatic Hydrolysis

#### 3.3.1. Enzyme Selection for HT Biotransformation

β-Glucosidase, hemicellulase, tannase, neutral protease, cellulase, glucoamylase, papain, alkaline protease, amylase and β-glucanase have different enzyme activities. Therefore, the same enzyme activity was used for OE hydrolysis to find the best enzyme. Olive leaf extract (81.04% OE, 500 mg) was put in 100 mL Erlenmeyer flasks, and the same enzyme activity (2.5 U/mg OE substrate) was also added. The hydrolysis conditions used were the best temperature and the best pH value of each enzyme for 6 h, respectively. Then the hydrolysates were all extracted by ethyl acetate and the rate of degradation of OE and HT content were determined with the following equations:

(2) The degradation rate of OE=1−(OE content in hydrolysate/OE content in olive leaf extract)

(3) The HT content=HT content in hydrolysate/weight of olive leaf extract)

#### 3.3.2. Substrate for HT Biotransformation

Olive leaf extract (38.6% OE, 1 g) was put in a 100 mL Erlenmeyer flask, then hemicellulase (50 mg) was also added. The enzymatic conditions were 60 °C, pH = 5, 0.1 mol/L phosphate buffer (50 mL) for 6 h. Then the hydrolysate was extracted by ethyl acetate.

Olive leaf extract (81.04% OE, 500 mg) was put in a 100 mL Erlenmeyer flask, then hemicellulase (50 mg) was also added. The enzymatic conditions were 60 °C, pH = 5, 0.1 mol/L phosphate buffer (50 mL) for 6 h. Then the hydrolysate was extracted by ethyl acetate.

#### 3.3.3. Single Factor Experimental Design

Olive leaf extract (81.04% OE, 500 mg) was put in a 100 mL Erlenmeyer flask, then hemicellulase (50 mg) was also added. The enzymatic conditions were 60 °C, pH = 5 phosphate buffer (50 mL) for 6 h. Then the hydrolysate was extracted by ethyl acetate. The effects of pH (4.0, 4.5, 5.0, 5.5, 6.0, 6.5, 7.0), temperature (30, 40, 50, 60, 70, 80 °C), time (1, 2, 3, 4, 5, 6, 7, 8, 9, 10 h), hemicellulase enzyme quantity (5, 10, 15, 20, 25, 50, 75, 100, 125, 150, 175, 200 mg) on HT content were studied by a single factor design as follows: one factor was changed while the other factors were kept constant in each experiment.

#### 3.3.4. Box-Behnken Design

The Box-Behnken design (BBD) is a novel analytical method for the optimization of processes. It presents the relationship between factors and involved responses during the optimization of analytical systems. In this work, BBD was used to predict the levels of factors pH (X_1_), temperature (X_2_), and enzyme quantity (X_3_). Table 6 showed the input parameters and experimental design levels used. The experimental design consisted of a set of points lying at the midpoint of each edge and the replicated center points of the multidimensional cube. The Design-Expert (version 7.1.3) statistical software (Stat-Ease, Minneapolis, MN, USA) was adopted to analyze the experimental data.

**Table 6:** Independent variables and their levels for Box-Behnken design of hydrolysis reaction.

Independent Variables	Symbol	Variable Levels		
		−1	0	1
pH	X_1_	4.5	5.0	5.5
Temperature (°C)	X_2_	40	50	60
Enzyme quantity (mg)	X_3_	25	50	75

### 3.4. High Performance Liquid Chromatography (HPLC) Analysis

The identification and quantification of phenolic compounds (OE and HT) were performed by HPLC. The equipment was a SPD-20A instrument (Shimadzu, Tokyo, Japan) equipped with a DAD detector (280 nm). The column was a 5 μm BDS HYPERSIL C18 (250 × 4.6 mm, Thermo, Boston, MA, USA), and its temperature was maintained at 30 °C. The mobile phase was 0.2% phosphoric acid in water (A) and methanol (B). The flow rate was 0.8 mL/min during a total running time of 20 min, and the injection volume was 10 μL. Identification of HT and OE were based on their HPLC spectra and their retention times in comparison with standards analysed under the same conditions. Furthermore, the quantifications of HT and OE were based on external calibration curves. The OE content had a good linear relationship with peak area in the range of 0.441–7.056 μg, and the regression equation was y = 1772846.78x + 89802.31 (R^2^ = 0.9997, y stands for peak area and x stands for OE content). The HT content had a good linear relationship with peak area in the range of 0.5586–8.9376 μg, and the regression equation was y = 1199063.90x + 119947.13 (R^2^ = 0.9996, y stands for peak area and x stands for HT content).

### 3.5. Total Phenol Content Determination

Content of total phenols was measured as gallic acid equivalents [29]. Diluted sample (1 mL) was transferred to a test tube; distilled water (6 mL) and Folin-Ciocalteu phenol reagent (1 mL) were added. Then 15% Na_2_CO_3_ (2 mL) was added. After the process, the 10 mL of total solution was well mixed and kept in the dark for 1 h. Then the samples were shaken and the absorbance was measured at 760 nm with an UV spectrophotometer.

### 3.6. DPPH (1,1-Diphenyl-2-Picrylhydrazyl) Radical Scavenging Assay

The DPPH radical-scavenging effect was evaluated according to Ni *et al*. [30]. Each diluted sample (1 mL) was added to a DPPH methanol solution (3 mL, 40 μg/mL). After the two solutions were gently mixed and left for 30 min at room temperature, the optical density was measured at 517 nm by a spectrophotometer. The antioxidant activity of each sample was expressed in terms of IC_50_ (microgrammes per mL required to inhibit DPPH radical formation by 50%) and calculated from the log-dose inhibition curve.

### 3.7. Statistical Analysis

Data were subjected to statistical analysis using Origin 7.5 software (OriginLab, Hampton, MA, USA). The data were reported as mean ± standard deviation (SD). The experiments were repeated three times. Differences were considered significant at *p* < 0.05.

## 4. Conclusions

In conclusion, single factor experiments and a Box-Behnken design was successfully employed to optimize the enzymatic hydrolysis parameters of OE. Additionally, the enzymatic hydrolysis results with different substrates (38.6% and 81.04% OE) were compared and the DPPH antioxidant properties were also evaluated. This study suggested the best hydrolysis treatment was using hemicellulase as a biocatalysts. The HT content of the 38.6% OE enzymatic hydrolysate was not higher than for 81.04% OE samples. Hydrolysis conditions were investigated with 81.04% OE olive leaf extract using hemicellulase. The optimal enzymatic conditions for achieving a maximal yield of HT content obtained by the regression were as follows: pH 5, temperature 55 °C and enzyme quantity 55 mg. Under these conditions, the experimental result was 11.31% ± 0.15%, which agreed closely with the predicted yield value (11.28%), and the degradation rate of OE was 98.54%. From the present investigation of antioxidant activity, the phenol content and radical scavenging effect were decreased after enzymatic hydrolysis by hemicellulase. However, the ethyl acetate extract of the enzymatic hydrolysate had high antioxidant activity (IC_50_ = 41.82 μg/mL). This might mean that the OE was partly decomposed into the OE aglycone, but not completely transformed into the HT by the enzymatic process.

## Abbreviation

HT: hydroxytyrosol
OE: oleuropein
BHT: butylated hydroxytoluene
DPPH: 1,1-diphenyl-2-picrylhydrazyl
BG: β-glucosidase
T: tyrosol
CHD: coronary heart disease
